# THE BORG SCALE IS SAFE AND EFFICACIOUS FOR PRESCRIBING AND MONITORING SELF-ADMINISTERED BALANCE AND RESISTANCE EXERCISE IN PATIENTS WITH CHRONIC KIDNEY DISEASE: A POST-HOC ANALYSIS OF RENEXC, A RANDOMIZED CONTROLLED TRIAL

**DOI:** 10.2340/jrm.v57.44158

**Published:** 2025-11-12

**Authors:** Philippa SVENSSON, Matthias HELLBERG, Anita WISÉN, Naomi CLYNE

**Affiliations:** 1 Department of Nephrology, Clinical Sciences Lund, Faculty of Medicine, Lund University and Skåne University Hospital, Lund; 2 Department of Health Sciences, Division of Rehabilitation & Sustainable Health, Faculty of Medicine, Lund University, Lund, Sweden

**Keywords:** exercise, postural balance, renal insufficiency, chronic, resistance training

## Abstract

**Objective:**

To evaluate the safety, adherence, and efficacy of self-administered balance and resistance exercise using the Borg scale in patients with chronic kidney disease, and to examine relationships between exercise intensity or duration and baseline measures, and relationships between change in physical performance and baseline measures, intensity, or duration.

**Design:**

Post-hoc analysis of a randomized controlled trial.

**Patients:**

151 patients, mean age 66 (14) years, measured glomerular filtration rate 22 (8) mL/min/1.73 m^2^.

**Methods:**

12 months balance or resistance exercise, Borg 13–17, combined with aerobic exercise. Berg Balance scale, Functional Reach, isometric quadriceps strength and 30 s sit-to-stand were assessed at baseline and after 12 months. Intensity and duration were recorded in an exercise diary.

**Results:**

No injuries occurred. Patients reported high adherence, median intensity 14 (13–15). Both groups maintained/improved physical performance by 2–18%, within a wide duration, mean 56 (range 3–194) min/week. No significant relationships were found between intensity or duration and baseline measures, or between improved physical performance and baseline measures, intensity, or duration.

**Conclusion:**

Twelve months’ self-administered balance and resistance exercise were safe and adhered to, using the Borg scale, in patients with chronic kidney disease. Physical performance improved, showing that even short weekly durations can be efficacious when prescribed intensity is maintained.

## LAY ABSTRACT

Exercise is important to prevent loss of balance and strength in patients with chronic kidney disease. In this study patients exercised independently at home or at a gym and were prescribed either balance or strength exercises by a physiotherapist. Exercise intensity was prescribed and monitored using the Borg scale of rating of perceived exertion. We found that exercise was safe to perform, resulted in high participation, and patients followed the prescribed exercise intensity, although there was a wide range of reported weekly exercise duration. Both groups improved balance and strength regardless of baseline measurements, showing that the Borg scale was safe and efficacious both for balance and resistance exercise in patients with chronic kidney disease, and that even short exercise durations resulted in improved physical performance when the prescribed exercise intensity was maintained.

Chronic kidney disease (CKD) affects around 10% of the global population (1), of whom a majority do not require kidney replacement therapy. Level of physical activity (2), balance, muscular strength, and aerobic capacity all decline with decreasing kidney function (3). Moreover, a low level of physical activity and poor physical performance are associated with an increased risk of mortality in these patients (4). There is convincing evidence of health benefits from physical activity in patients with CKD (3, 5–8), where current recommendations are to engage in moderate intensity physical activity for at least 150 min per week (9). To our knowledge, no studies have specifically examined the weekly intensity or duration of exercise needed to increase physical performance in the typical patient with CKD not on kidney replacement therapy, and few studies have examined balance exercise in patients with CKD (10, 11). The increasing number of patients with CKD indicates a need for a sustainable, self-administered exercise prescription, as patients often prefer exercising at home (12, 13). Although recent studies with longer intervention periods have examined home-based exercise interventions (14–20), little is known about the best methods to prescribe and monitor self-administered balance and resistance exercise programmes.

The Borg Rating of Perceived Exertion (RPE) scale (21) has mainly been used to prescribe intensity for aerobic exercise in patients with CKD (3), and some studies have used it for prescribing intensity for resistance exercise (17, 19, 22). While originally developed to correlate with heart rate (21), studies have since shown that using the Borg scale is useful as a subjective measure of exertion even in patients where heart rate does not reflect the absolute intensity, such as in patients with beta-blockers (23). The Borg RPE scale has been validated as a method evaluating exercise intensity in resistance exercise, where levels of exertion can be influenced by load, volume, proximity to failure, lactate accumulation etc. (24). However, quantifying exercise intensity for static and dynamic balance exercise is complex, and there is a lack of knowledge on how to best prescribe, monitor, and report exercise intensity for balance interventions (25). To our knowledge, there are no studies using the Borg Rating of Perceived Exertion scale to prescribe intensity in balance exercise in patients with CKD.

The primary aim of this study was to examine whether the Borg scale can be used to ensure safety, adherence, and efficacy during 12 months of self-administered balance and resistance exercise in patients with CKD. A secondary aim was to examine how RPE and weekly duration were associated with baseline measures, and to examine how change in balance, muscular strength, and muscular endurance were associated with baseline measures, RPE or weekly duration.

## METHODS

### Study design

This study is a post-hoc analysis of data from RENEXC (RENal EXerCise), a single-centre randomized controlled trial, in patients with CKD stages 3 to 5 not on kidney replacement therapy. Study design and primary data for the RENEXC trial have been thoroughly detailed previously (14) but baseline characteristics, comorbidity burden, and effects on physical performance are described again for clarity. The RENEXC trial compared 2 treatment arms: 1 group was randomized to perform balance exercise, and the other group was randomized to perform resistance exercise during a 12-month intervention period. Both groups performed additional aerobic endurance exercise (26). Assessment of balance, muscular strength, and muscular endurance, exercise prescription, and follow-up were performed by 2 research physiotherapists: p1 evaluated 54 baseline and 11 x 12-month measurements, and p2 evaluated all remaining measurements.

### Patients

Patients were recruited consecutively from the outpatient clinic of the Nephrology Department at Skåne University Hospital, Lund, Sweden. Inclusion criteria were no kidney replacement therapy, an estimated glomerular filtration rate < 30 mL/min/1.72 m^2^, ability to communicate in the Swedish language, and ≥ 18 years of age. Measured glomerular filtration rate (mGFR) was analysed after inclusion using iohexol clearance. Comorbidities were accepted to reflect clinical reality in the CKD population. Exclusion criteria were expected start of kidney replacement therapy within 1 year of study start, severe neurological or orthopaedic disorders making participation in self-administered exercise physically impossible, unstable cardiovascular disease, uncontrolled hypertension, and severe electrolyte disturbances.

### Comorbidity assessment

Comorbidities were assessed prior to inclusion using the Davies Comorbidity Score (27).

### Borg Rating of Perceived Exertion scale

The Borg scale is a subjective measure of exertion during physical activity, rating the perceived exertion based on a 6–20 scale, where 6 signifies no exertion and 20 represents maximal exertion (21). In this study, exercise intensity for balance and resistance exercise was prescribed, self-monitored, and adjusted using the Borg scale. As there is a lack of consensus on how to evaluate exercise intensity in balance exercise, we chose to use the Borg scale in order to quantify, adapt, and report balance exercise intensity. The patients were instructed to assess the level of challenge to maintain balance during the exercise using the verbal anchors of the Borg scale, e.g., a RPE of 13 corresponding to “somewhat hard”, to maintain balance throughout the static or dynamic balance exercise. To classify level of challenge, number of repetitions, duration, and degree of postural sway were evaluated. For dynamic exercises the range of motion was also evaluated.

### Exercise intervention

Patients were randomized to perform self-administered balance or resistance exercise, combined with aerobic endurance exercise, during a 12-month intervention period. Exercise intensity for balance and resistance exercise was prescribed within an RPE of 13–17 “somewhat hard” to “very hard” on the Borg scale, with the goal of reaching 90 min of balance or resistance exercise per week or more. All patients were prescribed additional aerobic endurance exercise within 13–15 “somewhat hard” to “hard” on the Borg scale, at 60 min/week. Balance and resistance exercises were individually prescribed regarding frequency, intensity, type, and duration based on randomization and the patient’s physical performance at baseline. Patient preference and goals were taken into consideration when designing the exercise programme. The self-administered exercise could be performed in the patient’s home or at a nearby gym according to the patient’s preference. If exercise was performed at home, the research physiotherapist gave instructions on how to perform the exercise programme at the outpatient clinic at Skåne University Hospital, and the patient was given dumbbells and weight cuffs to use at home. If the patient chose to perform exercise at a gym, the research physiotherapist gave instructions on how to perform the exercise programme on location, using the existing gym equipment. All exercise sessions were preceded by a warm-up.

Balance exercises were performed as 4–6 different exercises comprising both static and dynamic exercises. For static balance exercises, 1 repetition was equal to maintaining balance in a position for 30–60 s, repeated for 3–5 sets. Examples of static balance exercises used were maintaining balance while standing on one leg, standing with the feet together, standing in a tandem stance, standing on a balance board (single or double stance), planking, or maintaining balance in different positions using a Pilates ball. Dynamic exercises were performed as 10 repetitions/exercise and in 2–3 sets, and could include walking heel-to-toe, arm reaches while in a double- or single-leg stance, side-shuffles with turns, lunges with rotation, or single-leg dead lifts. Exercise intensity of the balance exercise programme could be raised by increasing the time for holding a static position, performing an exercise with eyes closed, integrating upper-body movement, adding light weights, gradually increasing the pace of the dynamic exercises, using an unstable surface, or by replacing the exercise with another of greater difficulty.

Resistance exercise was performed as 4–6 different exercises for 10–12 repetitions in 2–3 sets. Resistance programmes included dynamic exercises for the trunk and the upper and lower extremities and aimed to include a combination of larger and smaller muscle groups. Examples of resistance exercises used were quadriceps extensions, hamstrings curls, squats, step-ups, sit-ups, push-ups, back extensions, biceps curls, overhead press, lat-pulldowns, chest press, or dumbbell rows. The exercise intensity of the resistance programme could be raised by increasing the weights, changing the body position or by replacing one exercise with another of greater difficulty.

Follow-up and adjustment of the exercise programme regarding exercise intensity and duration was performed through phone contact, every week for the first 3 months and every other week for the remaining time of the intervention. To maintain exercise intensity within the predetermined RPE the intensity was increased corresponding to the patient’s progression and tolerance or reduced in the case of patient deterioration. Patients performed in-centre follow-up after 4 and 8 months of exercise, which allowed for added adjustment of exercise intensity and duration, as well as changing the exercises included in the exercise programme.

### Exercise diary

Patients noted the RPE for each individual exercise as well as the duration (in minutes) of each exercise session in an exercise diary. Patients were also able to comment on why they had not performed the exercise as planned. The exercise diary was sent to the research physiotherapist at regular intervals and mean RPE/week and mean min/week were calculated. The weekly or biweekly phone calls permitted completion of any missing data, enabling the limitations of a self-kept exercise diary to be taken into consideration (28, 29).

### Measures of balance, muscular strength, and muscular endurance

Measures of balance, muscular strength, and muscular endurance were assessed at baseline and after 4, 8, and 12 months of exercise.

*Berg Balance Scale*. The Berg Balance Scale (BBS) (30) is used to assess the patient’s ability to maintain balance during 14 predetermined tasks. Each task is scored on a scale of 0–4, where 0 indicates the lowest level of function (the patient is unable to perform the task) and 4 indicates the highest level of function (the patient is able to complete the task fulfilling the specified criteria assigned to it). The maximum score is 56 points. The BBS is a valid tool to measure balance in older adults (30) and has high reliability in different clinical populations (31), with minimal detectable change (MDC) between 2.8 and 6.6 points (32).

*Functional Reach*. Functional Reach (FR) (33) is a dynamic balance test. It measures the difference between arm’s length and maximal forward reach, using a fixed base of support. The test is performed in a standing position. The FR test has good reliability and validity in older adults (34). To our knowledge, no MDC has been established for FR in patients with CKD.

*Isometric quadriceps strength*. Isometric quadriceps strength (IQS) (35) was measured with a Salter scale (Salter, Oldham, UK) by extending the knee against resistance while in a sitting position. A mean value was calculated from 3 consecutive measurements for the right and left leg respectively. Measurements were given in kg x cm. This method has been used extensively in clinical practice in patients with CKD; however, there are no studies evaluating its psychometric properties.

*30 Second Sit-to-Stand Test*. The 30 Second Sit-to-Stand Test (STS30) (36) measures the number of stands a patient can complete during 30 s. It is a functional test, designed to assess strength and endurance in the lower extremity. The STS30 has high reliability and validity in patients with CKD, with an MDC of 2.1–2.6 repetitions (37).

### Harms

The patients were encouraged to contact the research physiotherapist in case of any exercise-related incidents, and any harms were assessed clinically at each follow-up or in-centre visit.

### Determination of sample size

This study was powered to detect at least a 10% improvement in muscular strength and muscular endurance, measured as isometric quadriceps strength and the STS30. To detect 60% differences at a standard deviation of 5% and 80% of power, we calculated that 75 patients per group were required to achieve complete data for 50 patients at the end of the intervention.

### Randomization and blinding

Patients were randomized using ProcPlan in SAS (SAS Institute, Cary, NC, USA). The block permutations and the size of each block were known by the statistician only, who had no contact with the patients, and the randomization process was designed so that it was not possible to predict which treatment would be assigned to a specific patient. The statistician generated the random allocation sequence, the nephrologist enrolled patients, and the physiotherapist assigned the intervention to the participants. Due to the nature of the intervention, blinding of the research physiotherapists and patients was not feasible.

### Statistical analysis

Data were analysed using R software (R Foundation for Statistical Computing, Vienna, Austria) using per protocol analysis. Descriptive statistics are presented with mean values ± standard deviations or median with interquartile range or range. Data distribution was assessed with the Shapiro–Wilk test. Changes in balance, muscular strength, and muscular endurance after 12 months of exercise were analysed with Student’s paired *t*-test for parametric data or with the Wilcoxon signed rank sum test for non-parametric data. To compare mean change in BBS, FR, IQS, and STS30 between the 2 treatment groups, the independent samples Student’s *t*-test was used. Relationships between dependent variables (RPE, weekly exercise duration [min/week]) and independent variables (baseline results in BBS, FR, IQS, and STS30) were analysed with single and multiple linear regression analyses, where adjusted models included the explanatory variables; age, sex, and change in mGFR.

Relationships between dependent variables (change in FR, IQS, and STS30) and independent variables (baseline results in FR, IQS, STS30, RPE, and exercise duration [min/week]) were analysed with single and multiple linear regression analyses, where adjusted models included the explanatory variables; age, sex, and change in mGFR. To account for the inflation of Type I error due to multiple testing, we applied the Benjamini–Hochberg false discovery rate procedure across all multiple regression models. Both raw and adjusted *p*-values are reported. *p*-values < 0.05 were considered significant. As balance measured by BBS was unchanged in both groups after 12 months of exercise, no relationships between change in BBS measurements and independent variables were analysed.

### Ethical considerations

The RENEXC trial was approved by the Regional Ethical Review Board in Lund (2011/369) and adhered to the Helsinki declaration. All patients received oral and written information and gave written informed consent. The RENEXC-trial is registered at www.ClinicalTrials.gov (NCT02041156. Registered on 17 January 2014).

## RESULTS

**Fig. 1:**
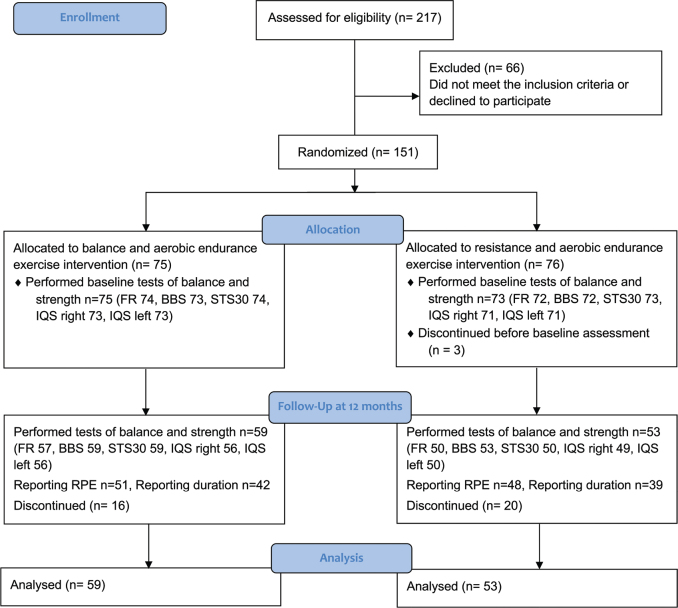
CONSORT flowchart of the RENEXC-trial.

Altogether, 217 patients were screened for eligibility, of whom 151 patients were included. Three patients were excluded from the analysis as they discontinued before performing the baseline measurements. A total of 148 patients performed measures of balance, muscular strength, and muscular endurance at baseline, 75 in the balance group and 73 in the resistance group. The included patients had a mean age of 65 (14) years and a mean mGFR of 23 (9) mL/min/1.73 m^2^ in the balance group, and a mean age of 67 (14) years and a mean mGFR of 22 (8) mL/min/1.73 m^2^ in the resistance group. Patients were recruited according to their eGFR. Once they joined the study GFR was measured, which explains the discrepancy between the inclusion criteria: eGFR< 30 mL/min/1.73 m^2^ and the actual results with mGFR. Participant recruitment began in October 2011 and concluded in May 2016. Follow-up was completed by May 2017. The trial ended after the final patient had completed 12 months of exercise. Baseline characteristics and comorbidities for each group are described in Table I. During the 12-month intervention period 16 patients in the balance group and 20 patients in the resistance group discontinued the study, as shown in the CONSORT flow diagram (Fig. 1).

**Table I:** Clinical characteristics at baseline.

Item	Balance group *n* = 75	Resistance group *n* = 73
Characteristics		
Age, years, mean (SD)	65 (14)	67 (14)
Sex, female/male, *n*	28/47	22/51
Weight, kg, mean (SD)	79 (16)	84 (19)
Height, m, mean (SD)	1.71 (0.1)	1.72 (0.09)
Body mass index, kg/m^2^, mean (SD)	27 (5)	28 (6)
mGFR, ml/min/1.73m^2^, mean (SD)	23 (9)	22 (8)
CKD stage 3, *n*	9	5
CKD stage 4, *n*	49	44
CKD stage 5, *n*	17	24
Comorbidities		
Malignancy, %	12	16
Diabetes mellitus, %	27	38
Left ventricular dysfunction, %	13	8
Ischaemic heart disease, %	17	22
Peripheral vascular disease, %	17	24
Systemic collagen vascular disease, %	9	12
Other significant pathology (e.g., hypertension), %	76	76

SD: standard deviation; mGFR: measured glomerular filtration rate.

Balance, muscular strength, and muscular endurance improved significantly in both groups (Table II) for FR (balance group: *p* = 0.007, resistance group: *p* = 0.003), IQS right and left (balance group: right *p* = 0.017, left *p* < 0.001, resistance group: right *p* < 0.001, left *p* = 0.016) and STS30 (balance group: *p* = 0.013, resistance group: *p* = < 0.001). Balance measured by BBS was unchanged in both groups. There were no significant between-group differences in mean change of balance and strength measures after 12 months of exercise (Table II).

**Table II:** Results for measures of balance, muscular strength, and muscular endurance at baseline and after 12 months of exercise.

Measure	Balance group				Resistance group				Group comparison
	*n*	Baseline	12 months	*p*-value	*n*	Baseline	12 months	*p*-value	*p*-value
BBS (points)	73	55 (51–56)	56 (53–56)	0.081^1^	72	54 (49–56)	56 (53–56)	0.287^1^	0.965
FR (cm)	74	34 (9)	36 (8)	**0.007**	72	32 (9)	36 (7)	**0.003**	0.948
IQS right leg (kg x cm)	73	1139.7 (410.1)	1213.8 (427.6)	**0.017**	71	1148.1 (410.1)	1332.0 (435.6)	**< 0.001**	0.283
IQS left leg (kg x cm)	73	1105.0 (417.5)	1192.6 (437.5)	**< 0.001**	71	1160.4 (426.4)	1305.2 (494.9)	**0.016**	0.864
STS30 (*n*)	74	12 (6)	13 (8)	**0.013**	73	11 (6)	13 (7)	**< 0.001**	0.449

BBS: Berg Balance Scale; FR: Functional Reach; IQS: Isometric Quadriceps Strength; STS30: 30 Second Sit-to-Stand Test. Values are presented as median (25th–75th percentile) for Berg Balance Scale or mean (standard deviation) for remaining measurements. *P*-value from Wilcoxon signed rank sum test or the paired *t*-test, *p*-values < 0.05 were considered significant. *P*-values for the group comparison of mean change after 12 months of exercise are from Student’s *t*-test, with *p*-values of < 0.05 considered significant. Significant values are highlighted in bold.

A total of 112 patients completed the study (59 patients in the balance group and 53 in the resistance group). Adherence to the prescribed RPE and weekly exercise duration during the 12-month study period is presented in Table III. Of the patients reporting RPE (balance group 71%, resistance group 74%), most patients reported an RPE within the prescribed interval of 13–17 (balance group: 83%, resistance group: 90%). Seven patients in the balance group and 3 in the resistance group reported a lower RPE than prescribed (< 13), where 6 patients reported RPE 12, 3 patients reported an RPE of 11 and 1 patient reported an RPE of 8.

**Table III:** Proportion of patients reporting RPE, duration and adherence to prescription of intensity and duration among the completers

	Balance group *n* = 59	Resistance group *n* = 53
Patients reporting RPE,* n* (%)	42 (71%)	39 (74%)
RPE, median (IQR)	14 (13–15)	14 (13–15)
Patients reached goal of RPE 13–17, *n* (%)	35 (59%)	36 (68%)
RPE/week < 13, *n* (%)	7 (12%)	3 (6%)
RPE/week > 17, *n* (%)	0 (0%)	0 (0%)
Patients reporting exercise duration, *n* (%)	51 (86%)	48 (91%)
Duration of exercise, minutes per week, median (IQR)	38, 3–194 (24–60)	62, 7–178 (35–81)
Patients reached goal of 90 minutes of exercise/week, *n* (%)	5 (8%)	11 (21%)

RPE: Rating of Perceived Exertion; IQR: interquartile range.

Exercise duration (min/week) was reported by 86% of the balance group and 91% in the resistance group. Mean duration was 38 (40) min/week for balance exercise and 62 (35) min/week for resistance exercise. There was a wide range of exercised min/week (balance group: 3–194, resistance group: 7–178). Only 8% in the balance group and 21% in the resistance group reached the target of 90 min or more per week.

No exercise-related incidents or injuries were reported during the continuous follow-up or in the self-kept exercise diary in either group.

**Fig. 2:**
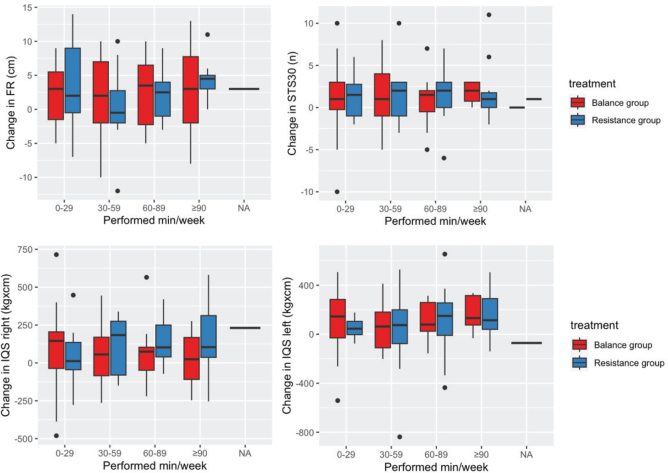
Change in Functional Reach (FR), isometric quadriceps strength (IQS), and 30 Second Sit to Stand Test (STS30) after 12 months of exercise at different durations of exercise (mean min/week).

No significant associations were observed between RPE, weekly exercise duration, and baseline measures after correcting for multiple comparisons, as presented in Table IV. No significant associations were observed between improvement in balance, muscular strength, and muscular endurance after 12 months of exercise and baseline results, or RPE after correcting for multiple comparisons, as presented in Table V. There were no significant associations between weekly duration and change in any of the measures in either the balance or the resistance group after 12 months of exercise after correcting for multiple comparisons (see Table V). Fig. 2 displays the distribution of change (Δ) in balance and strength measures in the balance and resistance group across different categories of weekly exercise duration. Across all duration groups, the distributions of Δ balance and Δ strength appear relatively similar, with no consistent trend or systematic shift observed.

**Table IV:** Multiple linear regression analyses of mean RPE/week and exercise duration (mean min/week) during 12 months of exercise and explanatory variables; baseline results in balance, muscular strength, and muscular endurance, age, sex, and deltaGFR after 12 months of exercise.

Dependent variable	Reported RPE Mean RPE/week						Reported exercise duration Mean min/week					
Group	Balance			Resistance			Balance			Resistance		
	B (95% CI)	*p*-value	Adjusted *p*-value	B (95% CI)	*p*-value	Adjusted *p*-value	B (95% CI)	*p*-value	Adjusted *p*-value	B (95% CI)	*p*-value	Adjusted *p*-value
BBS baseline	–0.01 (–0.08–0.06)	0.801	0.930	0.01 (–0.07–0.09)	0.824	0.929	–0.40 (–2.70–1.90)	0.736	0.899	–0.92 (–2.77–0.94)	0.338	0.776
Age	–0.07^***^ (–0.11 to –0.04)			0.02 (–0.03–0.06)			0.12 (–0.94–1.18)			0.10 (–0.85–1.05)		
Sex (male)	–0.22 (–0.99–0.56)			1.28^*^(0.08–2.48)			–5.71 (–29.15–17.72)			17.88 (–7.20–42.96)		
DeltaGFR	–0.003 (–0.06–0.06)			0.03 (–0.06–0.12)			1.22 (–0.50–2.93)			1.04 (–0.96–3.04)		
	Model fit: R² = 0.301, Adjusted R² = 0.236, F(4, 43) = 4.624, *p* = 0.003, Residual SE = 1.310			Model fit: R² = 0.145, Adjusted R² = 0.063, F(4, 42) = 1.777, *p* = 0.152, Residual SE = 1.695			Model fit: R² = 0.043, Adjusted R² = –0.036, F(4, 49) = 0.545, *p* = 0.704, Residual SE = 41.870			Model fit: R² = 0.100, Adjusted R² = 0.019, F(4, 44) = 1.228, *p* = 0.313, Residual SE = 37.650		
FR baseline	–0.00 (–0.06–0.05)	0.9780	0.978	0.09^*^ (0.02–0.16)	**0.015**	0.200	1.36 (–0.40–3.11)	0.137	0.457	0.10 (–1.54–1.74)	0.910	0.966
Age	–0.07^**^ (–0.11 to –0.03)			0.04 (–0.00–0.08)			0.80 (–0.43–2.03)			0.33 (–0.66–1.31)		
Sex (male)	–0.20 (–0.96–0.57)			0.95 (–0.20–2.09)			–6.24 (–28.96–16.49)			17.27 (–9.19–43.73)		
DeltaGFR	–0.00 (–0.06–0.05)			–0.00 (–0.09–0.08)			1.02 (–0.64–2.68)			0.67 (–1.32–2.67)		
	Model fit: R² = 0.297, Adjusted R² = 0.230, F(4,42) = 4.43, *p* = 0.004, Residual SE = 1.32			Model fit: R² = 0.257, Adjusted R² = 0.186, F(4, 42) = 3.62, *p* = 0.013, Residual SE = 1.58			Model fit: R² = 0.083, Adjusted R² = 0.008, F(4,49) = 1.11, *p* = 0.362, Residual SE = 4.97			Model fit: R² = 0.082, Adjusted R² = –0.002, F(4, 44) = 0.98, *p* = 0.430, Residual SE = 38.04		
IQS right baseline	0.00 (–0.00–0.00)	0.287	0.730	0.00 (–0.00–0.00)	0.129	0.502	–0.01 (–0.06–0.03)	0.587	0.899	0.01 (–0.03–0.05)	0.681	0.899
Age	–0.06^**^ (–0.10 to –0.02)			0.02 (–0.02–0.06)			–0.00 (–1.22–1.21)			0.36 (–0.55–1.27)		
Sex (male)	–0.60 (–1.67–0.48)			0.54 (–0.96–2.04)			0.61 (–30.54–31.76)			12.99 (–20.78–46.76)		
DeltaGFR	–0.00 (–0.06–0.06)			0.04 (–0.05–0.12)			1.16 (–0.56–2.88)			0.73 (–1.17–2.63)		
	Model fit: R² = 0.314, Adjusted R² = 0.247, F(4, 41) = 4.683, *p* = 0.003, Residual SE = 1.315			Model fit: R² = 0.190, Adjusted R² = 0.113, F(4, 42) = 2.464, *p* = 0.060, Residual SE = 1.649			Model fit: R² = 0.045, Adjusted R² = –0.034, F(4, 48) = 0.570, *p* = 0.686, Residual SE = 42.220			Model fit: R² = 0.085, Adjusted R² = 0.002, F(4, 44) = 1.019, *p* = 0.408, Residual SE = 37.970		
IQS left baseline	0.00 (–0.00–0.00)	0.379	0.794	0.00 (–0.00–0.00)	0.132	0.502	–0.01 (–0.05–0.03)	0.732	0.899	0.01 (–0.03–0.05)	0.619	0.899
Age	–0.06^**^ (–0.10 to –0.02)			0.03 (–0.02–0.07)			0.06 (–1.08–1.25)			0.39 (–0.55–1.33)		
Sex (male)	–0.50 (–1.53–0.52)			0.32 (–1.37–2.01)			–1.82 (–31.86–28.22)			10.43 (–27.65–48.51)		
DeltaGFR	–0.00 (–0.06–0.06)			0.04 (–0.05–0.12)			1.16 (–0.57–2.88)			0.74 (–1.16–2.64)		
	Model fit: R² = 0.310, Adjusted R² = 0.244, F(4, 42) = 4.714, *p* = 0.003, Residual SE = 1.314			Model fit: R² = 0.189, Adjusted R² = 0.112, F(4, 42) = 2.453, *p* = 0.061, Residual SE = 1.650			Model fit: R² = 0.042, Adjusted R² = –0.038, F(4, 48) = 0.523,* p* = 0.720, Residual SE = 42.300			Model fit: R² = 0.086, Adjusted R² = 0.003, F(4, 44) = 1.041, *p* = 0.397, Residual SE = 37.940		
STS baseline	0.03 (–0.05–0.10)	0.470	0.899	0.01 (–0.08–0.10)	0.877	0.961	0.44 (–1.93–2.80)	0.718	0.899	0.63 (–1.38–2.65)	0.540	0.899
Age	–0.06^**^ (–0.10 to –0.03)			0.01 (–0.03–0.06)			0.31 (–0.83–1.45)			0.41 (–0.52–1.35)		
Sex (male)	–0.19 (–0.94–0.57)			1.27^*^ (0.07–2.48)			–4.89 (–28.09–18.31)			16.54 (–8.98–42.05)		
DeltaGFR	–0.01 (–0.07–0.05)			0.03 (–0.05–0.12)			1.11 (–0.60–2.82)			0.60 (–1.33–2.52)		
	Model fit: R² = 0.308, Adjusted R² = 0.244, F(4, 43) = 4.79, *p* = 0.002, Residual SE = 1.303			Model fit: R² = 0.144, Adjusted R² = 0.063, F(4, 42) = 1.770, *p* = 0.153, Residual SE = 1.695			Model fit: R² = 0.043, Adjusted R² = –0.035, F(4, 49) = 0.549, *p* = 0.700, Residual SE = 41.860			Model fit: R² = 0.089, Adjusted R² = 0.006, F(4, 44) = 1.076, *p* = 0.380, Residual SE = 37.880		

* *p* < 0.05;

** *p* < 0.01;

*** p < 0.001, RPE: rating of perceived exertion, GFR: glomerular filtration rate, B: unstandardized coefficient, CI: confidence interval, BBS: Berg Balance Scale, FR: Functional Reach, STS30: 30 Second Sit-to-Stand Test, IQS: isometric quadriceps strength. Adjusted *p*-values from Benjamini–Hochberg false discovery rate procedure. *p*-values of <0.05 were considered significant, significant values are highlighted in bold. Models with *p* > 0.05 are considered non-significant.

**Table V:** Multiple linear regression analyses of change in balance, muscular strength, and muscular endurance after 12 months of exercise and the explanatory variables; baseline results in balance, muscular strength, and muscular endurance, RPE, exercise duration, age, sex and deltaGFR after 12 months of exercise (all dependent variables are delta values).

Dependent variable	FRw						IQS right						IQS left						STS30					
Group	Balance			Resistance			Balance			Resistance			Balance			Resistance			Balance			Resistance		
	B (95% CI)	p-value	Adjusted p-value	B (95% CI)	p-value	Adjusted p-value	B (95% CI)	p-value	Adjusted p-value	B (95% CI)	p-value	Adjusted p-value	B (95% CI)	p-value	Adjusted p-value	B (95% CI)	p-value	Adjusted p-value	B (95% CI)	p-value	Adjusted p-value	B (95% CI)	p-value	Adjusted p-value
Baseline results	–0.28^*^ (–0.52 to –0.05)	**0.021**	0.235	–0.35^**^ (–0.56 to –0.14)	0.002	0.088	–0.32^**^ (–0.53 to –0.11)	**0.004**	0.088	–0.11 (–0.31–0.09)	0.268	0.731	–0.17 (–0.35–0.01)	0.059	0.371	–0.08 (–0.26–0.09)	0.353	0.777	0.06 (–0.13–0.25)	0.535	0.899	0.19^*^ (0.01–0.37)	**0.043**	0.315
Age	–0.06 (–0.22–0.10)			–0.10 (–0.23–0.02)			–10.37^***^ (–16.10 to –4.64)			–1.70 (–7.24–3.84)			–6.05^*^ (–11.35 to –0.75)			–3.75 (–8.99–1.49)			–0.08 (–0.18–0.01)			0.04 (–0.04–0.12)		
Sex (male)	0.89 (–2.10–3.88)			–1.01 (–4.23–2.20)			185.21^*^ (37.85–332.57)			149.18 (–34.77–333.14)			130.65 (–6.69–267.98)			57.01 (–125.92–239.94)			–1.21 (–3.12–0.71)			–1.37 (–3.45–0.70)		
DeltaGFR	–0.03 (–0.25–0.18)			0.05 (–0.20–0.29)			9.97^*^ (1.86–18.07)			11.03 (–0.25–22.31)			4.35 (–3.44–12.15)			23.25^***^ (12.54–33.96)			0.00 (–0.14–0.14)			0.04 (–0.12–0.20)		
	Model fit: R² = 0.128, Adjusted R² = 0.054, F(4, 47) = 1.726, *p* = 0.160, Residual SE = 5.264			Model fit: R² = 0.269, Adjusted R² = 0.194, F(4, 39) = 3.589, *p* = 0.014, Residual SE = 4.404			Model fit: R² = 0.237, Adjusted R² = 0.170, F(4, 46) = 3.566, *p* = 0.013, Residual SE = 200.9			Model fit: R² = 0.155, Adjusted R² = 0.068, F(4, 39) = 1.781, *p* = 0.152, Residual SE = 198.7			Model fit: R² = 0.193, Adjusted R² = 0.108, F(4, 38) = 2.275, *p* = 0.079, Residual SE = 195.8			Model fit: R² = 0.345, Adjusted R² = 0.280, F(4, 40) = 5.270, *p* = 0.002, Residual SE = 188.3			Model fit: R² = 0.142, Adjusted R² = 0.072, F(4, 49) = 2.028, *p* = 0.105, Residual SE = 3.417			Model fit: R² = 0.127, Adjusted R² = 0.040, F(4, 40) = 1.458, *p* = 0.233, Residual SE = 2.966		
RPE /week	–0.12 (–1.46–1.22)	0.918	0.962	–0.79 (–1.72–0.13)	0.102	0.499	1.73 (–46.50–49.97)	0.944	0.966	8.35 (–34.50–51.21)	0.704	0.899	–28.11 (–72.86–16.64)	0.225	0.707	7.75 (–31.83–47.32)	0.703	0.899	0.18 (–0.63–0.98)	0.664	0.899	0.16 (–0.51–0.82)	0.645	0.899
Age	0.07 (–0.11–0.24)			0.01 (–0.13–0.15)			–4.82 (–10.91–1.27)			0.02 (–5.68–5.72)			–5.27 (–11.07–0.53)			–3.78 (–9.09–1.53)			–0.07 (–0.17–0.04)			–0.01 (–0.09–0.08)		
Sex (male)	0.30 (–3.09–3.69)			–0.76 (–4.64–3.12)			–10.53 (–133.80–112.75)			40.70 (–132.32–213.71)			16.27 (–99.76–132.30)			–36.61 (–196.47–123.26)			–1.61 (–3.65–0.43)			–1.54 (–4.22–1.14)		
DeltaGFR	–0.04 (–0.29–0.22)			–0.03 (–0.30–0.24)			13.97^**^ (4.70–23.24)			12.01 (0.05–23.97)			5.76 (–2.95–14.47)			23.40^***^ (12.26–34.53)			0.03 (–0.12–0.18)			0.04 (–0.14–0.22)		
	Model fit: R² = 0.025, Adjusted R² = –0.072, F(4, 40) = 0.261, *p* = 0.901, Residual SE = 5.718			Model fit: R² = 0.109, Adjusted R² = 0.010, F(4, 36) = 1.104, *p* = 0.370, Residual SE = 4.794			Model fit: R² = 0.250, Adjusted R² = 0.177, F(4, 41) = 3.414, *p* = 0.017, Residual SE = 208.8			Model fit: R² = 0.126, Adjusted R² = 0.029, F(4, 36) = 1.298, *p* = 0.289, Residual SE = 207.2			Model fit: R² = 0.122, Adjusted R² = 0.036, F(4, 41) = 1.424, *p* = 0.243, Residual SE = 196.3			Model fit: R² = 0.345, Adjusted R² = 0.274, F(4, 37) = 4.870, *p* = 0.003, Residual SE = 193.1			Model fit: R² = 0.132, Adjusted R² = 0.049, F(4, 42) = 1.595, *p* = 0.193, Residual SE = 3.442			Model fit: R² = 0.041, Adjusted R² = -0.063, F(4, 37) = 0.395, *p* = 0.811, Residual SE = 3.229		
Duration, min/week	0.01 (–0.04–0.05)	0.805	0.929	0.05^*^ (0.01–0.10)	**0.031**	0.279	–0.80 (–2.24–0.64)	0.283	0.731	1.62 (–0.28–3.51)	0.102	0.499	0.70 (–0.61–2.02)	0.299	0.731	1.21 (–0.60–3.01)	0.198	0.670	0.01 (–0.02–0.03)	0.615	0.899	0.01 (–0.02–0.04)	0.429	0.858
Age	0.05 (–0.08–0.19)			–0.08 (–0.22–0.06)			–5.01 (–10.04–0.03)			–1.54 (–7.22–4.14)			–3.50 (–8.13–1.14)			–4.79 (–10.22–0.63)			–0.10^*^ (–0.19 to –0.02)			–0.01 (–0.10–0.08)		
Sex (male)	0.50 (–2.65–3.64)			–4.07^*^ (–7.61 to –0.54)			32.50 (–87.70–152.70)			29.48 (–116.64–175.59)			52.22 (–57.62–162.05)			–25.53 (–164.33–113.27)			–1.26 (–3.20–0.68)			–1.40 (–3.74–0.94)		
DeltaGFR	–0.07 (–0.30–0.16)			–0.07 (–0.34–0.20)			10.91^*^ (2.00–19.83)			11.67^*^ (0.58–22.76)			3.66 (–4.47–11.79)			23.09^***^ (12.49–33.69)			0.01 (–0.14–0.15)			0.05 (–0.13–0.22)		
	Model fit: R² = 0.024, Adjusted R² = –0.059, F(4, 47) = 0.292, *p* = 0.881, Residual SE = 5.569			Model fit: R² = 0.176, Adjusted R² = 0.089, F(4, 38) = 2.030, *p* = 0.110, Residual SE = 4.735			Model fit: R² = 0.192, Adjusted R² = 0.124, F(4, 47) = 2.799, *p* = 0.036, Residual SE = 214.6			Model fit: R² = 0.193, Adjusted R² = 0.108, F(4, 38) = 2.275, *p* = 0.079, Residual SE = 195.8			Model fit: R² = 0.102, Adjusted R² = 0.025, F(4, 47) = 1.329, *p* = 0.273, Residual SE = 195.9			Model fit: R² = 0.359, Adjusted R² = 0.294, F(4, 39) = 5.468, *p* = 0.001, Residual SE = 187.2			Model fit: R² = 0.139, Adjusted R² = 0.067, F(4, 48) = 1.932, *p* = 0.120, Residual SE = 3.457			Model fit: R² = 0.047, Adjusted R² = -0.051, F(4, 39) = 0.479, *p* = 0.751, Residual SE = 3.138		

* *p* < 0.05;

** *p* < 0.01;

*** *p* < 0.001, RPE: rating of perceived exertion, GFR: glomerular filtration rate, B: unstandardized coefficient, CI: confidence interval, FR: Functional Reach, IQS: isometric quadriceps strength, STS30: 30 Second Sit-to-Stand Test. Adjusted *p*-values from Benamini–Hochberg false discovery rate procedure. *p*-values of <0.05 were considered significant, significant values are highlighted in bold. Models with *p* > 0.05 are considered non-significant.

## DISCUSSION

This head-to-head RCT comparing self-administered balance and resistance exercise showed that the Borg RPE scale was a safe and efficacious method in patients with CKD 3–5 over a 12-month period. A main finding was that significant improvements in balance, muscular strength, and muscular endurance were achieved by both exercise regimes, irrespective of number of minutes of exercise per week. Of note, our study is one of the first to examine the use of the Borg scale in this context, giving a unique input on how safe and efficacious exercise protocols can be implemented in patients with CKD.

The prescribed RPE of 13–17 was achievable regardless of the patient’s baseline status, with no significant relationships between RPE and baseline measures. Weekly exercise duration showed no significant relationships with baseline measures in either group. While duration was reported by most completers, ranging from 3–194 min/week, few met the 90 min/week target (8% in the balance group and 21% in the resistance group). Factors such as comorbidities, motivation, or habitual exercise levels might explain the wide range of exercise duration.

Our results showed that patients could achieve improvements in balance, muscular strength, and muscular endurance through self-administered balance or resistance exercise, regardless of baseline status, as we found no significant relationships between improvements and baseline measures of BBS, FR, IQS, and STS30. While research in the general population has shown that deconditioned individuals reap the greatest relative benefits of resistance exercise on physical performance (38), this was not observed in our study.

Both groups improved physical performance with a consistent RPE, and a wide variation of duration, demonstrating efficacy even at shorter weekly durations. Interestingly, no significant relationship was found between improvements in balance, muscular endurance, or muscular strength, and RPE or weekly duration of exercise in either group, suggesting that improvements were achieved when RPE was kept within the prescribed interval, irrespective of exercise duration. This is in accordance with results found for aerobic endurance exercise in a previous post-hoc analysis of the RENEXC trial (26). Most patients reported an RPE between 13 and 15 (interquartile range), suggesting that this is a sufficient level of exertion or challenge to achieve an improvement. Similarly, the EXCITE trial in patients on dialysis demonstrated that low-volume exercise interventions can result in meaningful improvements, measured with the 6-Minute Walk test and 5 Times Sit-to-Stand Test (15), as well as a lower risk of hospitalization (39). A systematic review (40) found that health benefits can be achieved at half the recommended exercise volume of 150 min/week or less, especially when transitioning from sedentary behaviour to exercise, supporting individualized exercise prescription to prevent barriers through a one-size-fits-all approach (7, 40). Given many patients’ limited tolerance of prolonged exercise, our findings support the use of shorter, manageable exercise sessions that include both balance and strength and involve multiple muscle groups. This approach can minimize fatigue, improve adherence, and help patients incorporate physical activity into their routine without feeling overwhelmed.

Our results demonstrated high adherence to the 12-month intervention, reinforcing the feasibility of self-administered exercise in patients with CKD, consistent with a previous meta-analysis (41). Key factors include individualized exercise programmes and adaptation to maintain the target RPE, regular follow-up, and support from the multidisciplinary team (42). As illustrated in Fig. 2, improvements in balance and strength were found across a range of weekly durations with few achieving the target duration. In consequence, maintained or improved balance and strength can be achieved by an individualized exercise prescription, taking into account physical performance, prior exercise experience, and motivation. Given the high prevalence of comorbidities and frailty in this population, an individualized exercise programme and progression plan can overcome barriers such as fatigue and low physical performance (42). The absence of exercise-related incidents in our study further supports the safety of this approach, aligning with results from other exercise trials in patients with CKD (15–17, 19, 41).

Our study has several strengths, with broad inclusion criteria reflecting real-life patients, as well as being one of the few studies to date that has examined self-administered exercise over a 12-month period in patients with CKD and with a high adherence rate. However, there are some limitations. While the Borg RPE scale was developed to evaluate exertion in aerobic exercise (21) and has been validated to evaluate exercise intensity in resistance exercise (24), there are no studies examining the Borg scale to evaluate intensity in balance exercise. Exercise intensity in balance exercise is seldom reported in trials (25) and there are no clear recommendations in the literature on how to estimate exercise intensity in balance exercise. Therefore, we chose to extend the application of the Borg scale to balance exercise, using the verbal anchors to describe “levels of challenge” rather than levels of exertion, thus facilitating communication between the research physiotherapist and patient and enabling adaptation of the exercise intensity through phone contact. As our results showed high adherence in reporting exercise intensity with the Borg scale, and most patients reached the prescribed exercise intensity as well as significantly improving balance and strength, our findings suggest that the Borg scale could be useful also for balance exercise; however, additional research is required to confirm these findings.

Although both groups showed a statistically significant improvement in FR, IQS, and STS30, the only test evaluated for MDC in patients with CKD is STS30, for which improvement did not exceed the MDC. Nevertheless, considering the expected disease-related decline of balance and strength in this group of patients, maintaining or slightly improving BBS, FR, IQS, and STS30 is deemed to be clinically relevant. Furthermore, the Berg Balance Scale showed a ceiling effect, indicating the need for more sensitive measures in future research, and using instruments with established psychometric properties to evaluate IQS would enhance the interpretability and generalizability of the findings. Reliance on self-reported exercise data may have been subject to recall bias and reporting bias, which could be improved in future studies by including objective measures such as wearable activity trackers. Additionally, the research physiotherapist’s dual role in prescribing exercise and assessing the patients’ physical performance might have contributed to detection bias. Both physiotherapists adhered to a standardized protocol, thus minimizing risk of bias. The lack of a sedentary control group limits comparative interpretation, but including a sedentary control group was deemed unethical as prescription of exercise was part of standard care in our department. Despite these limitations, this is the first study to compare 2 different exercise programmes in patients with CKD 3–5, with high adherence during the 12-month study period, providing valuable insights on how self-administered exercise can be prescribed and monitored for patients with CKD.

In conclusion, 12 months of self-administered balance and resistance exercise using the Borg RPE scale 13–17 was safe and adhered to in patients with CKD 3–5. Critical to the intervention’s success were individualized exercise prescriptions, adaptive progression, and multidisciplinary support. Patients improved all aspects of physical performance within a wide range of exercised minutes per week, showing that even short weekly durations can be highly efficacious if prescribed RPE is maintained.

## Trial details

The RENEXC-trial is registered at www.ClinicalTrials.gov (NCT02041156. Registered on 17 January 2014).

Data available on request. Trial protocol and statistical analysis plan available on request.
